# Krill oil attenuates left ventricular dilatation after myocardial infarction in rats

**DOI:** 10.1186/1476-511X-10-245

**Published:** 2011-12-29

**Authors:** Linn E Fosshaug, Rolf K Berge, Jan O Beitnes, Kjetil Berge, Hogne Vik, Pål Aukrust, Lars Gullestad, Leif E Vinge, Erik Øie

**Affiliations:** 1 Department of Internal Medicine, Diakonhjemmet Hospital, Oslo, Norway; 2 Research Institute for Internal Medicine, Oslo University Hospital Rikshospitalet, Oslo, Norway; 3 Centre for Heart Failure Research, University of Oslo, Oslo, Norway; 4 Section of Medical Biochemistry, Institute of Medicine, University of Bergen, Bergen, Norway; 5 Department of Heart Disease, Haukeland University Hospital, Bergen, Norway; 6 Department of Cardiology, Oslo University Hospital, Rikshospitalet, Oslo, Norway; 7 Aker BioMarine ASA, Oslo, Norway; 8 Section of Clinical Immunology and Infectious Diseases, Oslo University Hospital Rikshospitalet, Oslo, Norway

**Keywords:** Heart failure, n-3 polyunsaturated fatty acids, lipids

## Abstract

**Background:**

In the western world, heart failure (HF) is one of the most important causes of cardiovascular mortality. Supplement with n-3 polyunsaturated fatty acids (PUFA) has been shown to improve cardiac function in HF and to decrease mortality after myocardial infarction (MI). The molecular structure and composition of n-3 PUFA varies between different marine sources and this may be of importance for their biological effects. Krill oil, unlike fish oil supplements, contains the major part of the n-3 PUFA in the form of phospholipids. This study investigated effects of krill oil on cardiac remodeling after experimental MI. Rats were randomised to pre-treatment with krill oil or control feed 14 days before induction of MI. Seven days post-MI, the rats were examined with echocardiography and rats in the control group were further randomised to continued control feed or krill oil feed for 7 weeks before re-examination with echocardiography and euthanization.

**Results:**

The echocardiographic evaluation showed significant attenuation of LV dilatation in the group pretreated with krill oil compared to controls. Attenuated heart weight, lung weight, and levels of mRNA encoding classical markers of LV stress, matrix remodeling and inflammation reflected these findings. The total composition of fatty acids were examined in the left ventricular (LV) tissue and all rats treated with krill oil showed a significantly higher proportion of n-3 PUFA in the LV tissue, although no difference was seen between the two krill oil groups.

**Conclusions:**

Supplement with krill oil leads to a proportional increase of n-3 PUFA in myocardial tissue and supplement given before induction of MI attenuates LV remodeling.

## Background

In the western world, heart failure (HF) is one of the most important causes of cardiovascular mortality and myocardial infarction (MI) constitutes a major etiologic factor precipitating HF [1]. The molecular and cellular pathological processes that ultimately lead to HF are collectively referred to as cardiac remodeling and are characterized by cardiomyocyte hypertrophy, ventricular dilatation, and development of myocardial fibrosis [2].

Metabolic alterations also occur during development of HF with the hallmark change being a shift from myocardial oxidation of fatty acids (FA) to utilization of glucose as the main source of energy generation. Since metabolism of glucose requires less oxygen, this shift may be beneficial for the heart [3,4]. However, plasma FA may still constitute an important source of energy in HF, and it has been suggested that cardiac accumulation of lipids in HF can result in lipotoxicity and therefore contributes to the detoriation of cardiac function [4]. However, these issues are far from clear and even though some FA may be considered harmful, others, like n-3 polyunsaturated FA (PUFA), have been shown to decrease mortality after MI and in HF [5-7]. It has also been suggested that the composition of FA bound to the plasma membrane phospholipids may be of importance to myocardial function. In line with this notion, it has been shown that the pro-inflammatory membrane component arachidonic acid (AA) can be replaced with eicosapentaenoic acid (EPA) or docosahexaenoic acid (DHA) with an increased dietary intake of these n-3 PUFA [8].

Krill (*Euphausia superba*) is a small Antarctic crustacean and its extracted oil contains a high proportion of n-3 PUFA bound to phospholipids. This molecular makeup is different from traditional fish oils, where the n-3 PUFA are mainly bound to triglycerides or ethyl esters [9]. This difference may be important as the molecular form of n-3 PUFA has been suggested relevant for their biological effects [10]. Furthermore, phospholipids themselves have been shown to have beneficial effects on lipid metabolism [11]. These properties could suggest a beneficial effect of krill oil during MI and post-MI remodeling. To further elucidate this issue, we investigated the effects of krill oil on cardiac remodeling and function in rats after MI.

## Results

### Effect of krill oil on cardiac structure and function

There were no significant differences in tibia length (TL) and increase of body weight (BW) after 8 weeks between the MI groups. However, the heart weight-to-body weight and the lung weight-to-body weight ratios was significantly smaller in the MI-krill oil pretreated (PT) group compared to the MI-control and MI-krill oil non-pretreated (nPT) groups at 8 weeks (Table 1).

**Table 1:** Effect of krill oil on heart and lung weights and cardiac structure and function at baseline (before MI) and 1 and 8 weeks after induction of MI

		Control feed		Krill oil feed	
		
		Sham	MI	MI-nPT	MI-PT
Baseline:	BW (g)	273 ± 3	261 ± 3^#^	271 ± 2	270 ± 2*
1 week:	LVEDD (mm)	5.0 ± 0.2	8.1 ± 0.2^###^	7.9 ± 0.2	8.3 ± 0.2
	PWT (mm)	2.4 ± 0.1	2.2 ± 0.1^#^	2.3 ± 0.1	2.2 ± 0.1
	FS (%)	68 ± 2	21 ± 1^###^	22 ± 1	21 ± 1
	RWT	2.5 ± 0.1	2.2 ± 0.1^###^	2.3 ± 0.2	2.2 ± 0.1
8 weeks:	BW (g)	427 ± 7	397 ± 9^#^	427 ± 8*	425 ± 6*
	TL (mm)	38 ± 0.2	38 ± 0.4	38 ± 0.2	38 ± 0.3
	HW/BW (mg/g)	2.7 ± 0.2	3.7 ± 0.2^##^	3.6 ± 0.2	3.1 ± 0.1*^†^
	LW/BW (mg/g)	3.2 ± 0.1	6.2 ± 0.5^##^	6.3 ± 0.7	4.3 ± 0.5**^†^
	LVEDD (mm)	6.0 ± 0.2	10.5 ± 0.3^###^	11.1 ± 0.3	9.8 ± 0.3^**††**^
	PWT (mm)	3.1 ± 0.2	2.3 ± 0.1^##^	2.3 ± 0.1	2.4 ± 0.1
	FS (%)	69 ± 3	18 ± 1^###^	16 ± 1	18 ± 1
	RWT	3.1 ± 0.2	2.3 ± 0.1^###^	2.3 ± 0.1	2.4 ± 0.1

MI, myocardial infarction; nPT, non-pretreated; PT, pretreated; BW, body weight; TL, tibia length; HW, hearth weight; LW, lung weight; LEVDD, left ventricular diastolic diameter; PWT, posterior wall thickness; RWT, relative wall thickness; FS, fractional shortning. Values are mean ± SEM. ^#^P ≤ 0.05 vs sham, ^##^P ≤ 0.01 vs sham, ^###^P ≤ 0.001 vs sham, *P ≤ 0.05 vs MI-control, **P ≤ 0.01 vs MI-control, ***P ≤ 0.001 vs MI-control, ^**†**^P ≤ 0.05 vs MI-nPT, ^**††**^P ≤ 0.01 vs MI-nPT, ^**†††**^P ≤ 0.001 sv MI-nPT.

**Figure 1:**
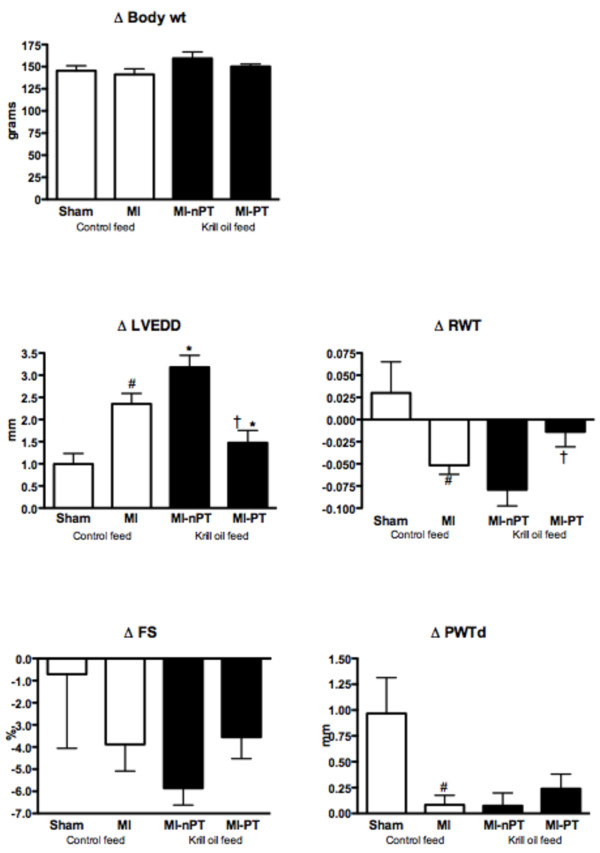
**Effect of krill oil on change in myocardial function and structure post-MI**. MI, myocardial infarction; nPT, non-pretreated; PT, pretreated; BW, body weight; LVEDD, left ventricular diastolic diameter; PWT, posterior wall thickness; RWT, relative wall thickness; FS, fractional shortning.^#^P ≤ 0.05 vs sham *P ≤ 0.05 vs MI-control. ^**†**^P ≤ 0.05 vs MI-nPT.

As expected, there was a significant increase in left ventricular (LV) end-diastolic diameter (LVEDD) in the MI-control group compared to the sham group (Figure 1). More importantly, the MI-krill oil PT group showed significantly less LV dilatation during the treatment period compared to both the MI-control and the MI-krill oil nPT group. An increase in LVEDD was seen in the MI-krill oil nPT group compared to the MI-control group. There were no differences in the change of posterior wall thickness (PWT) or fractional shortening (FS) between the different MI groups. However, the change in relative wall thickness (RWT) was significantly lower in the MI-krill oil PT group, compared to the MI-krill oil nPT group.

### Effects of krill oil on plasma lipids

Several significant alterations in plasma lipid levels were observed (Table 2). There were no significant differences in plasma lipids between the two krill oil groups, except that high-density lipoprotein (HDL)-cholesterol were lower in the MI-krill oil PT group compared to the MI-krill oil nPT group. However, when comparing the two krill oil groups to the MI-control group, the krill oil treated groups showed significantly reduced levels of total-, free- and low-density lipoprotein (LDL)-cholesterol as well as phospholipids and free FA (FFA) in plasma. Furthermore, levels of triglycerides were significantly higher in the MI-krill oil PT group than in the MI-control group.

**Table 2:** Effect of krill oil on plasma lipid concentrations 8 weeks after MI

	Control feed		Krill oil feed	
	
	Sham	MI	MI-nPT	MI-PT
	mmol/L			
Total cholesterol	2.09 ± 0,14	1.93 ± 0.08	1.73 ± 0.10*	1.59 ± 0.06**
HDL cholesterol	1.31 ± 0.07	1.33 ± 0.06	1.22 ± 0.06	1.05 ± 0.04***^†^
LDL cholesterol	0.24 ± 0.04	0.25 ± 0.02	0.20 ± 0.02*	0.16 ± 0.02**
Free cholesterol	0.56 ± 0.04	0.49 ± 0.02^#^	0.42 ± 0.02*	0.40 ± 0.01**
Triglycerides	1.67 ± 0.24	1.12 ± 0.17	1.53 ± 0.14*	1.59 ± 0.11**
Phospholipids	2.19 ± 0.12	1.91 ± 0.06^#^	1.75 ± 0.07	1.73 ± 0.05*
FFA	0.15 ± 0.04	0.34 ± 0.05^#^	0.12 ± 0.03**	0.11 ± 0.02***
Glucose	11.8 ± 0.47	11.6 ± 0.39	11.1 ± 0.37	10.8 ± 0.41

MI, myocardial infarction; nPT, non-pretreated; PT, pretreated; HDL, high density lipoprotein; LDL, low density lipoprotein; FFA, free fatty acids. Values are mean ± SEM. ^#^P ≤ 0.05 vs sham, *P ≤ 0.05 vs MI-control, **P ≤ 0.01 vsMI-control, ***P ≤ 0.001 vs MI-control, ^**†**^P ≤ 0.05 vs MI-nPT.

### Effects of krill oil on myocardial FA composition

There were no regional myocardial differences (non-infarcted vs border zone tissue) in the composition of FA in any of the groups (data not shown). As a consequence, the more elaborate analyses were performed only on non-infarcted tissue. Whereas the total amount of FA were unaffected upon induction of MI, the relative level of saturated FA (SFA) and n-3 PUFA were slightly elevated in the MI-control group when compared to the sham group. However, compared to the MI-control group, treatment with krill oil resulted in a higher myocardial proportion of n-3 PUFA and a lower relative level of n-6 PUFA, resulting in an increased n-3/n-6 PUFA ratio (Table 3). EPA (20:5 n-3) and DHA (22:6 n-3), both documented to be beneficial after MI and in HF in humans, were proportionally increased in the krill oil-treated groups. There were no differences in n-3 PUFA, n-6 PUFA, EPA, or DHA between the MI-krill oil PT and MI-krill oil nPT groups.

**Table 3:** Effect of krill oil on myocardial fatty acid composition 8 weeks after MI

	Control feed		Krill oil feed	
	
	Sham	MI	MI-nPT	MI-PT
	μg FA/g tissue			
Total FA	25240 ± 1342	24911 ± 3701	22585 ± 1912*	23935 ± 1205^**†**^
	wt.% of total FA			
Palmitic acid	9.41 ± 0.25	11.07 ± 0.38^##^	11.72 ± 0.11***	11.78 ± 0.11***
EPA	0.18 ± 0.01	0.18 ± 0.01	1.58 ± 0.07***	1.75 ± 0.06***
DPA	2.04 ± 0.06	2.40 ± 0.09^#^	3.62 ± 0.08***	3.61 ± 0.08***
DHA	8.98 ± 0.41	10.6 ± 0.44^#^	16.06 ± 0.03***	15.77 ± 0.29***
AA	22.9 ± 0.42	23.77 ± 0.7^#^	17.16 ± 0.32***	14.37 ± 0.29***^†††^
SFA	30.4 ± 0.25	31.8 ± 0.16^###^	32.0 ± 0.17	31.3 ± 0.15^**†**^
MUFA	8.03 ± 0.33	8.8 ± 0.77	8.36 ± 0.22	8.8 ± 0.18
PUFA n-3	11.62 ± 0.44	13.6 ± 0.44^##^	21.59 ± 0.27***	21.5 ± 0.31***
PUFA n-6	49.72 ± 0.59	45.6 ± 0.58^###^	37.89 ± 0.30***	38.2 ± 0.38***
PUFA n-3/n-6	0.24 ± 0.01	0.30 ± 0.01^###^	0.57 ± 0.01***	0.6 ± 0.01***

MI, myocardial infarction; nPT, non-pretreated; PT, pretreated; wt.%, weight percent; FA, fatty acids; EPA, eicosapentaenoic acid; DPA, docosapentaenoic acid; DHA, docosahexaenoic acid; AA, arachidonic acid; SFA, saturated FA; MUFA, monounsaturated FA; PUFA, polyunsaturated FA. Values are mean ± SEM. ^#^P ≤ 0.05 vs sham, ^##^P ≤ 0.01 vs sham, ^###^P ≤ 0.001 vs sham, *P ≤ 0.05 vs MI-control, ***P ≤ 0.001 vs MI-control, ^**†**^P ≤ 0.05 vs MI-nPT, ^**†††**^P ≤ 0.001 vs MI-nPT.

### Alterations of genes involved in cardiac remodeling

Significantly attenuated levels of mRNA encoding classical markers of LV stress and matrix remodeling were observed in the MI-krill oil PT rats compared to the MI-control rats (Table 4). The same observation was not seen in the nPT rats. Furthermore, the mRNA levels of classical inflammatory mediators, like interleukin (IL)-6, were significantly lower in the MI-krill oil PT group compared to both the MI-control and MI-krill oil nPT groups.

**Table 4:** Alterations on genes involved in cardiac remodeling 8 weeks after MI

	Control feed		Krill oil feed	
	
	Sham	MI	MI-nPT	MI-PT
	Relative units			
ANP	0.17 ± 0.05	2.08 ± 0.35^###^	2.48 ± 0.36	1.21 ± 0.19*^††^
MMP-2	0.59 ± 0.13	2.44 ± 0.65^#^	1.88 ± 0.39	2.41 ± 0.53
MMP-9	0.07 ± 0.02	0.35 ± 0.20^##^	1.28 ± 0.69	1.68 ± 1.30
TIMP	0.74 ± 0.05	1.71 ± 0.18^#^	1.60 ± 0.24	1.05 ± 0.14*^†^
CTGF	0.28 ± 0.01	0.94 ± 0.01	0.87 ± 0.15	1.27 ± 0.19
TGF-β	0.95 ± 0.14	1.83 ± 0.22^#^	1.90 ± 0.20	1.37 ± 0.11^**†**^
TNF-α	1.39 ± 0.30	0.89 ± 0.20	2.24 ± 0.33**	0.99 ± 0.14^**††**^
IL-1β	0.89 ± 0.15	0.80 ± 0.10	1.11 ± 0.14	0.66 ± 0.07^**††**^
IL-6	0.88 ± 0.38	6.91 ± 2.38^##^	4.37 ± 0.83	1.92 ± 0.30*^†^
MCP-1	1.20 ± 0.43	2.43 ± 0.53	3.00 ± 0.54	1.19 ± 0.10^**††**^

Values are mean ± SEM. ^#^P ≤ 0.05 vs sham, ^##^P ≤ 0.01 vs sham, ^###^P ≤ 0.001 vs sham, *P ≤ 0.05 vs MI-control **P ≤ 0.01 vs MI-control, ^**†**^P ≤ 0.05 vs MI-nPT, ^**††**^P ≤ 0.01 vs MI-nPT.

## Discussion

In the present study, we show that treatment with krill oil prior to induction of MI attenuates ventricular dilatation and hypertrophy. These findings were further reflected by attenuated increase in lung weight, heart weight, and altered expression of various genes encoding peptides known as markers and mediators of myocardial remodeling. However, when treatment with krill oil was introduced 7 days after MI, increased ventricular dilatation was seen.

Since the beneficial effects of krill oil were dependent on initiation of treatment before induction of MI, our data indicate that krill oil may have a favorable influence on the initial remodeling process after MI. This hypothesis is supported by previous experimental studies demonstrating that n-3 PUFA given prior to aortic banding protects against ventricular remodeling and dysfunction in rats [8,12-15]. In the GISSI-HF trial, significant reductions in overall mortality and hospital admissions in patients with chronic HF were seen in patients receiving n-3 PUFA in the form of ethyl esters [6]. In a sub-study of GISSI-HF, the effects of n-3 PUFA on LV structure and function were investigated, and a small but significant effect on LV ejection fraction was observed [16]. In addition, a small study in patients with non-ischemic cardiomyopathy demonstrated increased LV ejection fraction after 12 months of supplementation with 2 g of n-3 PUFA [17]. However, no effects on LV structure were found, which support our hypothesis that krill oil may have beneficial effects on the initial cardiac remodeling process after MI rather than on the remodeling process in the more chronic phases after MI.

The rats pretreated with krill oil showed attenuated expression of several mRNA known to be involved in the cardiac remodeling process; tissue inhibitor of matrix metalloproteinase (TIMP), atrial natriuretic peptide (ANP), and IL-6. Several studies have shown that the balance of matrix metalloproteinase (MMP) and their endogenous inhibitors TIMP is an important regulator of ventricular dimensions as they regulate structure and function of the extracellular matrix (ECM) [18]. Myocardial ANP levels have been observed to increase in response to hemodynamic overload [19]. Also, elevated levels of inflammatory cytokines, like IL-6, have been observed in several studies on HF and cardiac remodeling [20]. In consequence of the assumed involvement of TIMP, ANP, and IL-6 in the regulation of the ECM structure and function, lower expression of these mRNA levels in the myocardial tissue could potentially be related to the echocardiographic finding of less LV dilatation in the rats pretreated with krill oil.

The beneficial effect of pretreatment with krill oil on post-infarction cardiac remodeling may also be a result of a favorable effect on MI size. Previous experimental studies have observed that n-3 PUFA induces significant myocardial resistance to ischemia-reperfusion injury and thereby significant smaller myocardial infarct size in rodents [21,22]. We therefore hypothesize that pretreatment with krill oil, in addition to possible effects on early cardiac remodeling, may also lead to smaller MI with less LV dilatation and hypertrophy. However, in the present study we have no data on MI size, and this hypothesis will have to be further explored in forthcoming studies.

The potential beneficial effects of krill oil on post-MI remodeling could have several explanations. First, as shown in our study, dietary supplement with n-3 PUFA leads to slightly reduced myocardial SFA levels. Substituting PUFA for SFA may be associated with lower risk of coronary heart disease and studies on isolated myocardial cells have shown that the SFA palmitic acid induces cardiomyocyte apoptosis under certain conditions [23,24]. Second, krill oil supplementation induced an increase in myocardial levels of EPA and DHA, which have been shown to be incorporated into cell membranes in both healthy and failing hearts [14,25]. This may lead to an increased production of anti-inflammatory or resolving mediators including resolvins of the E series. In contrast to n-3 PUFA, n-6 PUFA may lead to enhanced generation of inflammatory mediators including prostaglandin-E2 and lipoxins. As a consequence, a relative decrease in n-6 PUFA during krill oil supplementation may further enhance the anti-inflammatory properties of krill oil [8]. Finally, incorporation of n-3 PUFA into mitochondrial membranes under conditions of myocardial stress has also been hypothesized to be beneficial as it may help maintain myocardial oxidative function [26].

Even though this study was not designed as to compare the effects of fish oil to that of krill oil, other studies allows hypothesizing that the molecular form of the supplemented n-3 PUFA is of importance to their various effects [10]. It was recently demonstrated that the incorporation of EPA and DHA into myocardial phospholipids was higher when delivered in the form of krill oil/n-3 phospholipids, compared to n-3 bound in the form of triglycerides (fish oil) [27]. Two recent studies compared the absorption of DHA and EPA from triglycerides versus phospholipid n-3 PUFA sources and they showed that supplementation with krill oil gave a dose-equivalent higher plasma concentration of EPA and DHA in women and men compared to fish oil [28,29].

## Conclusions

In conclusion, although relatively few rats were studied, our findings may suggest that treatment with krill oil before MI leads to attenuated LV dilatation and hypertrophy in rats. However, future studies are needed to establish whether these beneficial effects are consequences of attenuated cardiac remodeling or reduction of MI sizes. Also, the molecular effects of krill oil on the heart are not yet clear and need to be examined further.

## Methods

### Rat model of MI and treatment protocol

**Figure 2:**
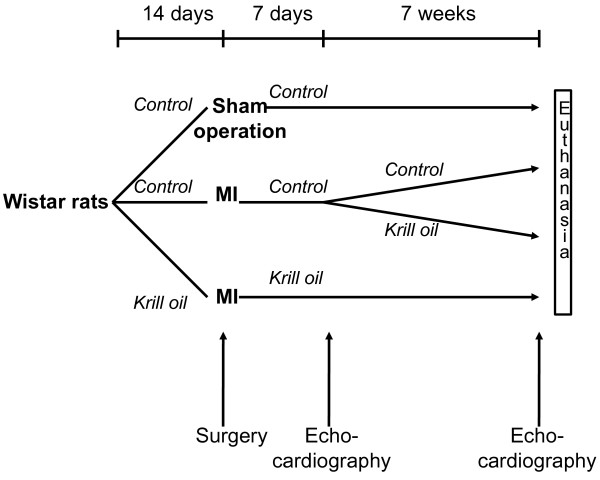
**Experimental rat model and treatment protocol**. Rats with transmural MIs were randomized in to three groups: 1) Krill oil prior to and after MI, 2) a control diet prior to and after induction of MI, or 3) a control diet prior to and 7 days after induction of MI, thereafter krill oil. Surviving rats were killed 8 weeks after the surgical procedure. In addition, a sham-operated group of rats were included on a normal feed for the entire length of the study.

One week prior to induction of MI, male Wistar rats (n = 173) were randomized in to two groups; 1) Pretreatment (PT) with Superba™ krill oil (Aker BioMarine ASA, Oslo, Norway) in the feed (n = 45), and 2) control feed (n = 128) (Figure 2). The rats underwent induction of MI by left coronary artery ligation as previously described [30]. A group of rats on control feed (n = 8) were sham-operated for comparison. Surviving rats (n = 97) were evaluated by transthoracic echocardiography at 7 days post-MI and only rats with transmural infarctions were included in the study (n = 53). The MI-rats pretreated with krill oil (n = 18) were then continued on the same diet and rats on control feed were further randomized to: 1) Treatment with krill oil (n = 17) and 2) control feed (n = 14). Treatment was continued for 7 weeks before reevaluation with transthoracic echocardiography and harvesting of the organs. Mortality was low during the treatment period: MI-krill oil PT group, 1 rat; MI-krill oil nPT group, 4 rats; MI-krill oil group, 0 rats; Sham group, 0 rats. The amount of EPA+DHA in 100 g of feed was 0.47 g, which is equivalent to 0.75% of the energy in the rat diet which corresponds to 1.67 g EPA+DHA/day in a 8.4-MJ/day diet in humans (Table 5). The "Principle of laboratory animal care" (NIH publication No. 86-23, revised 1985) was followed as well as Norwegian national laws regarding animal care.

**Table 5:** Fatty acid composition (grams pr 100 g diet) of the feeds as determined from reference values for the soy and krill oils mixed into the feeds

Fatty acid	Control diet	Krill oil diet
	**g/100 g diet**	
Palmitic acid	0.68	0.68
Stearic acid	0.29	0.20
Oleic acid	1.46	1.12
Linoleic acid	3.27	2.12
Alpha-linolenic acid	0.44	0.30
EPA	0	0.30
DHA	0	0.17
Arachidonic acid	0.01	0.01
SFA	1.02	1.00
MUFA	1.48	1.18
PUFA n-3	0.44	0.83
PUFA n-6	3.27	2.12
PUFA n-3/n-6	0.13	0.39

FA, fatty acids; EPA, eicosapentaenoic acid; DHA, docosahexaenoic acid; SFA, saturated FA; MUFA, monounsaturated FA; PUFA, polyunsaturated FA.

### Echocardiography

Echocardiography was performed prior to randomization 7 days after the surgical procedure and after 7 weeks of treatment (8 weeks post-surgery). The rats were sedated with 0.3 ml/100 g body wt Hypnorm^®^-Dormicum^® ^[2.5% Hypnorm^® ^(VetaPharma; fentanyl 0.315 mg/ml + fluanisone 10 mg/ml) + 25% Dormicum^® ^(Roche; midazolam 5 mg/ml) in water]. Transthoracic echocardiography was performed with a Vivid 7 scanner (GE) and a 14-MHz transducer. Two-dimensional-directed M-Mode echocardiography was obtained in the parasternal short-axis view at the level of the papillary muscles. The various echocardiographic parameters were calculated as follows: FS = LVEDD-LV end-systolic diameter (LVESD)/LVEDD)] × 100; RWT = PWT in diastole/LV internal radius (radius = 0.5 × LVEDD).

### Blood and tissue sampling

At the end of the study, blood was drawn from the abdominal aorta into EDTA-containing tubes and centrifuged within 20 min at 4°C (2000*g *for 20 min), plasma was aspirated, and then stored at -80°C until further analyses. The heart and lungs were weighed, and the tibia lengths were measured. The LV (septum and free wall) was separated from the rest of the heart and further sectioned into infarcted area, non-infarcted area, and the border zone between these two regions (approximately width of 2 mm). The tissue was immediately frozen in liquid nitrogen and stored at -80°C until further analysis.

### Isolation of total RNA, reverse transcription and real-time PCR analysis

Total RNA was extracted with acid-buffered phenol (Trizol^®^, Invitrogen, San Diego, CA), DNase treated, and further purified using RNeasy mini columns (Qiagen, Hilden, Germany). cDNA was prepared using the High-Capacity cDNA Archive Kit (Applied Biosystems, Foster City, CA) according to the manufacturer's instruction. Primer sequences were designed using the Primer Express software version 3.0 (Applied Biosystems, Foster City, CA), sequence information can be provided on request. Quantitative real-time PCR analysis was performed using an ABI Prism 7300 sequence detector (Applied Biosystems) and SYBR Green assays (qPCR Master Mix for SYBR Green I, Eurogentec, Seraing, Belgium). The ribosomal RNA P0 was used as an internal standard for normalization of target mRNA.

### Lipid analysis

Plasma lipids were measured on the Hitachi 917 system (Roche Diagnostics GmbH, Mannheim, Germany) using the following kits: total cholesterol (Bayer, Tarrytown, NY), free cholesterol (Wako Chemicals, Dalton, OH), HDL-cholesterol (HDL-C Plus, Roche Diagnostics GmbH), LDL-cholesterol (LDL-C Plus, Roche Diagnostics GmbH), triglycerides (Bayer), and phospholipids (PAP 150, bioMérieux, Lyon, France).

### Free FA and total levels and composition of FA

Plasma free FA (FFA) were measured on the Hitachi 917 system (Roche Diagnostics) using a commercially available FFA kit (NEFA [non-esterified FA] C, Wako Chemicals, Neuss, Germany). Measurement of total levels and composition of FA was performed after extracting lipids from the myocardial tissue using a mixture of chloroform and methanol. The extracts were transesterified using BF_3_-methanol. To remove neutral sterols and non-saponifiable material, extracts of fatty acyl methyl esters were heated in 0.5 M KOH in ethanol-water solution (9:1). Recovered FA were re-esterified using BF_3_-methanol. The methyl esters were quantified by gas chromatography as previously described [31].

### Statistical analysis

All data are presented as mean ± SEM. For comparisons of two groups, the Mann-Whitney U test was used. For comparison of over 3 groups, ANOVA with the Kruskal-Wallis test was used. If the Kruskal-Wallis revealed significant differences, subsequent analyses of individual means were performed with Mann-Whitney U test. A value of P < 0.05 was considered statistically significant. SPSS 18.0 was used for all analyses.

## Abbreviations

AA: arachidonic acid; ANP: atrial natriuretic peptide; BW: body weight; CTGF: connective tissue growth factor; DPA: docosapentaenoic acid; ECM: extracellular matrix; FA: fatty acid; FS: fractional shortening; HF: heart failure; HW: heart weight; LV: left ventricular; LVEDD: LV end-diastolic diameter; LVESD: LV end-systolic diameter; LW: lung weight; MCP: monocyte chemoattractant protein; MI: myocardial infarction; MMP: matrix metalloproteinase; nPT: non-pretreated; PT: pretreated; PWT: posterior wall thickness; RWT: relative wall thickness; TIMP: tissue inhibitor of MMP; TL: tibia length.

## Competing interests

HV and KB are employed by and the study was partially financed by Aker BioMarine ASA, Oslo, Norway. LEF, RKB, JOB, PA, LG, LEV, and EØ have no disclosures.

## Authors' contributions

All authors designed research. L.E.F., R.B., J.O.B, L.E.V., and E.Ø. conducted research. L.E.F. analyzed data and wrote the paper. L.E.F, L.E.V, and E.Ø. had primary responsibility for the final content. All authors read and approved the final manuscript
